# First determination of DNA virus and some additional bacteria from *Melophagus ovinus* (sheep ked) in Tibet, China

**DOI:** 10.3389/fmicb.2022.988136

**Published:** 2022-09-06

**Authors:** Yong-Hong Liu, Yi-Min Ma, Hong-Ou Tian, Bo Yang, Wen-Xiong Han, Wei-Hong Zhao, Hai-Liang Chai, Zhan-Sheng Zhang, Li-Feng Wang, Lei Chen, Yu Xing, Yu-Lin Ding, Li Zhao

**Affiliations:** ^1^College of Veterinary Medicine, Inner Mongolia Agricultural University, Hohhot, China; ^2^Key Laboratory of Clinical Diagnosis and Treatment Technology in Animal Disease, Ministry of Agriculture and Rural Affairs, Hohhot, China; ^3^Animal Disease Control Center of Ordos, Ordos City, China; ^4^Inner Mongolia Saikexing Reproductive Biotechnology (Group) Co., Ltd., Hohhot, China; ^5^Shanghai Origingene Bio-pharm Technology Co., Ltd., Shanghai, China

**Keywords:** *Melophagus ovinus*, sheep ked, Tibet, microbial population, viral metagenomics

## Abstract

*Melophagus ovinus* (sheep ked) is one of the common ectoparasites in sheep. In addition to causing direct damage to the host through biting and sucking blood, sheep ked is a potential vector of helminths, protozoa, bacteria, and viruses. Sheep *M. ovinus* samples from three regions in Tibet were selected for DNA extraction. The 16S rDNA V3-V4 hypervariable region was amplified, after genomic DNA fragmentation, Illumina Hiseq libraries were constructed. The 16S rRNA sequencing and viral metagenomics sequencing were separately conducted on the Illumina Novaseq 6000 platform and molecular biology software and platforms were employed to analyze the sequencing data. Illumina PE250 sequencing results demonstrated that the dominant bacteria phylum in *M. ovinus* from Tibet, China was Proteobacteria, where 29 bacteria genera were annotated. The dominant bacterial genera were *Bartonella*, *Wolbachia*, and *Arsenophonus*; *Bartonella chomelii*, *Wolbachia* spp., and *Arsenophonus* spp. were the dominant bacterial species in *M. ovinus* from Tibet, China. We also detected *Kluyvera intermedia*, *Corynebacterium maris* DSM 45190, *Planomicrobium okeanokoites*, and *Rhodococcus erythropolis*, of which the relative abundance of *Kluyvera intermedia* was high. Illumina Hiseq sequencing results demonstrated that 4 virus orders were detected in *M. ovinus* from Tibet, China, and 3 samples were annotated into 29 families, 30 families, and 28 families of viruses, respectively. Virus families related to vertebrates and insects mainly included Mimiviridae, Marseilleviridae, Poxviridae, Ascoviridae, Iridoviridae, Baculoviridae, Hytrosaviridae, Nudiviridae, Polydnaviridae, Adomaviridae, Asfarviridae, Hepeviridae, Herpesviridae, and Retroviridae; at the species level, the relative abundance of Tupanvirus_soda_lake, Klosneuvirus_KNV1, and Indivirus_ILV1 was higher. African swine fever virus and many poxviruses from the family Poxviridae were detected, albeit their relative abundance was low. The dominant bacterial phylum of *M. ovinus* from Tibet, China was Proteobacteria, and the dominant bacterial genera were Bartonella, Wolbachia, and Arsenophonus, where 23 out of 29 annotated bacteria genera were first reported in *M. ovinus*. *Kluyvera intermedia*, *Corynebacterium maris* DSM 45190, *Planomicrobium okeanokoites*, and *Rhodococcus erythropolis* were detected for the first time. All DNA viruses detected in this study have been reported in *M. ovinus* for the first time.

## Introduction

There is a type of wingless fly that has dense setae on the body surface and three pairs of legs with claws and belongs to the genus *Melophagus* and family Hippoboscidae (Diptera: Hippoboscoidea), which is a hematophagous ectoparasite of animals (Small, 2005). Its scientific name is *Melophagus ovinus* (*M. ovinus*) and the proper name is sheep ked (Ulicsni et al., 2016). *M. ovinus* is also known as ked or louse fly (Tang et al., 2018), and it is called by different folk names in different regions (such as uhkullancs [sheep kullancs] and kullancs) (Ulicsni et al., 2016). *M. ovinus* is commonly wrongly named as sheep tick (Small, 2005). Sheep is the permanent host of *M. ovinus* (Halos et al., 2004; Small, 2005; Kumsa et al., 2012; Liu et al., 2018, 2019; Zhao et al., 2018, 2020), but *M. ovinus* is also found in goats (Bequaert, 1942; Seyoum et al., 2015), rabbits (Small, 2005), dogs (Tetley, 1958), donkeys (Flores-Mendoza et al., 2021), European bison (Izdebska, 2001), red foxes (Lassnig et al., 1998), Tibetan sheep (Hao et al., 2020), Tibetan antelope (Liu et al., 2018; Zhao et al., 2018, 2020) and humans (Tetley, 1958). *M. ovinus* has a broad geographical distribution and is reported in Europe [e.g., Czechia (Chrudimský et al., 2012; Nováková et al., 2015; Rudolf et al., 2016), Hungary (Hornok et al., 2011), Slovakia (Hubálek et al., 1986), Croatia (Martinković et al., 2012), St Kilda (Gibson et al., 2010), France (Halos et al., 2004), Poland (Werszko et al., 2021), Russia (Litov et al., 2021)], Africa [e.g., Ethiopia (Kumsa et al., 2012, 2014; Seyoum et al., 2015; Hadgu et al., 2021), Algeria (Boucheikhchoukh et al., 2019), Kenya (Liu et al., 2016)], Oceania [e.g., Australia (Liu et al., 2016; Litov et al., 2021), New Zealand (Liu et al., 2016)], North America [e.g., United States (Halos et al., 2004; Kosoy et al., 2004, 2016)], South America [e.g., Peru (Flores-Mendoza et al., 2021)] and Asia [e.g., Turkey (Payzin, 1953), Mongolia, India, Japan (Liu et al., 2016)]. In recent years, *M. ovinus* has frequently appeared in China, Asia in Tibet (Chu et al., 2011), Gansu (Duan et al., 2017, 2020; Tang et al., 2018; Zhao et al., 2020), Xinjiang (Liu et al., 2016, 2017, 2019; Zhao et al., 2018, 2020), Sichuan (Hao et al., 2020), and Qinghai (Zhang et al., 2021).

*Melophagus ovinus* causes damage to the host *via* biting and blood-sucking in two aspects. On the hand, it causes restlessness, anemia, weight loss, wool loss, skin irritation and pruritus, skin damage, and reduced skin value in the host, which further causes inflammation, secondary microbial infections, cutaneous myiasis (Small, 2005), and scabies (No Author List., 2000), and decreases meat, milk, and fur yields in the host (Small, 2005). More importantly on the other hand, *M. ovinus* can carry or spread pathogens such as *Rickettsia* spp. (Hornok et al., 2011), *R. raoultii*, *R. slovaca* (Liu et al., 2016), *R. melophagi* (Arkwright et al., 1919; Cowdry, 1923; Hertig and Wolbach, 1924; Kligler and Aschner, 1931; Rudolf et al., 2016), *Bartonella* spp. (Halos et al., 2004; Reeves et al., 2006; Kumsa et al., 2014; Duan et al., 2020; Werszko et al., 2021), *Ba. melophagi* (Halos et al., 2004; Kosoy et al., 2004, 2016; Bemis and Kania, 2007; Kumsa et al., 2014; Rudolf et al., 2016; Liu et al., 2018; Boucheikhchoukh et al., 2019; Flores-Mendoza et al., 2021), *Ba. melophagi* variants variants, *Ba. chomelii* (Duan et al., 2020), *Ba. schoenbuchensis* (Halos et al., 2004), *Wolbachia* sp. (Duan et al., 2017, 2020; Liu et al., 2018; Boucheikhchoukh et al., 2019), *Trypanosoma* spp. (Werszko et al., 2021), *Tr. melophagium* (Hoare, 1923, 1972; Gibson et al., 2010; Martinković et al., 2012), *Tr. theodori* (Small, 2005), *Anaplasma ovis* (Zaugg and Coan, 1986; Hornok et al., 2011; Zhao et al., 2018; Zhang et al., 2021), *An. phagocytophilum*, *An. bovis* (Zhang et al., 2021), *Acinetobacter* spp. (Kumsa et al., 2012; Duan et al., 2017), *Ac. lwoffii* (Kumsa et al., 2012), *Borrelia burgdorferi* sensu lato (Werszko et al., 2021), *Bo. garinii*, *Bo. spirochetes* belonging to *Bo. valaisiana*-related group (Chu et al., 2011), *Theileria Luwenshuni* (Hao et al., 2020), *Th. ovis* (Zhao et al., 2020; Zhang et al., 2021), *Anaplasmataceae* family (Boucheikhchoukh et al., 2019), *Arsenophonus* spp. (Duan et al., 2017, 2020), *Ar. melophagi* (Husnik et al., 2020), Candidatus *Ar. melophagi* (Nováková et al., 2015), *Coxiella burnetii* (Pavilanis et al., 1958), Candidatus *Sodalis melophagi* (Chrudimský et al., 2012; Nováková et al., 2015; Boyd et al., 2016), *Streptococcus hyointestinalis*, *Lactobacillus amylovorus*, *L. johnsonii*, *L. intestinalis*, *L. salivarius*, *L. murinus*, *L. agilis*, *Escherichia coli*, *Enterococcus cecorum*, *Megasphaera elsdenii*, *Helicobacter ganmani*, *Clostridium leptum*, *Clostridium sp.* Culture 27, *Actinobacillus minor*, *Aureococcus anophagefferens*, *Dorea ormicigenerans*, *Lachnospiraceae bacterium* A2, *Eubacterium coprostanoligenes*, *Blautia coccoides* (Duan et al., 2020), *Pseudomonas aeruginosa*, *Shewanella* spp., *Sh. algae*, *Staphylococcus* spp., *St. xylosus*, *Enterobacter* spp., *Halomonas* spp., *Bacillus* spp. (Duan et al., 2017; Bezerra-Santos and Otranto, 2020). There are very few reports on *M. ovinus*-related viruses, which are all RNA viruses, such as blue-tongue virus (Luedke et al., 1965), Border disease virus (Liu et al., 2019), dengue virus (Setién et al., 2017), Khandagaity Melophagus iflfla-like virus, Bayan-Khairhan-Ula Melophagus solemo-like virus, Ulaatai Melophagus solemo-like virus, Aksy-Durug Melophagus sigmavirus, Berke-Baary Melophagus reo-like virus (Litov et al., 2021). In addition, *M. ovinus* from different regional sources were tested as negative for several specific pathogens, including those that were reportedly positive for *M. ovinus* in other regions (Dungal, 1946; Payzin, 1953; Nelder et al., 2008; Rudolf et al., 2016; Duan et al., 2017; Boucheikhchoukh et al., 2019; Flores-Mendoza et al., 2021; Werszko et al., 2021; Zhang et al., 2021). It is well-known that microorganisms in ectoparasites are unstable and that the microbiome composition and abundance are affected by the geographical origin and blood-feeding behavior. Therefore, there is a need for a comprehensive analysis of the diversity of microorganisms carried by *M. ovinus* from different regions.

For a long period, Hippoboscid flies (Diptera: Hippoboscidae) have been ignored by the scientific community, and its role as a vector of pathogens for humans and domestic animals has been scantly investigated; as a result, there is a lack of scientific knowledge on its biology, epidemiology as well as vector competence (Bezerra-Santos and Otranto, 2020). Examining the pathogens carried by *M. ovinus* in livestock or wild animals can help analyze the role of *M. ovinus* in epidemiology. High-throughput sequencing is expedient and efficient for the analysis of the microbial community structure of *M. ovinus*. However, high-throughput sequencing has been conducted only on microbial population and RNA virome in *M. ovinus* from Gansu province in China (Duan et al., 2017, 2020) as well as the Republic of Tuva in Russia (Litov et al., 2021) so far. However, to date, no study has employed modern metaviromics techniques to reveal the DNA virome of *M. ovinus*, and no study has conducted specific tests, such as PCR, to detect DNA viruses in *M. ovinus*. In this study, the Illumina Novaseq 6000 platform was employed to sequence *M. ovinus* from Tibet, China so as to determine the microbial community structure and diversity and analyze the DNA virome of *M. ovinus* in order to provide a foundation for predicting emerging pathogens, evaluating potential risks, preventing insect-borne diseases, and developing biological control tools.

## Materials and methods

### *Melophagus ovinus* collection

**FIGURE 1:**
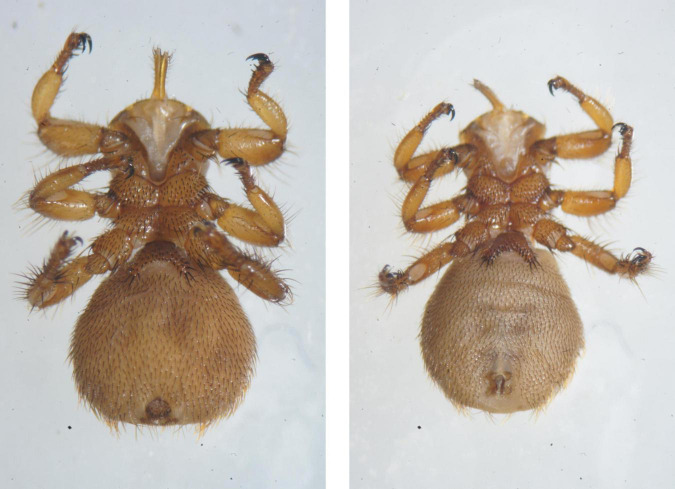
Photomicrographs of *Melophagus ovinus* (Left: Ventral view of the female; Right: Ventral view of the male).

*Melophagus ovinus* (Figure 1) was collected from Garze County in Rikaze, Tibet (4029 m above sea level; 28°91′N, 89°60′E) and Lhari County in Nagqu, Tibet (4,498 m above sea level; 30°63′N, 93°24′E) from April to May 2020. and from Nyingchi, Tibet (3,008 m above sea level; 29°57′N, 94°48′E) in July 2019. The hosts were all sheep, and the samples were subjected to identification using a combination of morphological and molecular biology methods. The sample was stored at −80°C. For the convenience of description, the abbreviations used to represent each sample are SK1, SK2, and SK3.

### DNA extraction and high-throughput sequencing

Four *M. ovinus* (two of each gender) were collected from each sampling point and mixed as one sample. The body surfaces of the insects in every sample were cleaned thrice with 70% ethanol and sterile deionized water. Next, TaKaRa MiniBEST Universal Genomic DNA Extraction Kit Ver. 5.0 (Takara, Beijing, China, Code No. 9765) was used to extract total DNA from the samples according to the manufacturer’s protocol in a biosafety cabinet, and the extracted DNA was stored at −20°C.

One aliquot of each sample, which served as a DNA template, and specific primers with barcodes were used to amplify the 16S rDNA V3-V4 hypervariable region using the TransStart FastPfu DNA Polymerase (TransStart^®^, Beijing, China, Code No. AP221-02) kit. The universal primers were 338F: 5′-ACT CCT ACG GGA GGC AGC A-3′, 806R: 5′-GGA CTA CHV GGG TWT CTA AT-3′. The PCR reaction volume was 25 μL, and the annealing temperature was 55°C. PCR products were recovered using the AXYPREP DNA Gel Extraction Kit (AXYGEN, Suzhou, China, Code No. AP-GX-50G). After the samples were subjected to 2% agarose gel electrophoresis, Illumina PE250 libraries were constructed using the VAHTS^®^ ssDNA Library Prep Kit (Illumina, San Diego, CA, United States, Code No. ND6201). Subsequently, 16S rRNA sequencing was conducted on the Illumina Novaseq 6000 platform (San Diego, CA, United States).

An additional portion of DNA from each sample was subjected to genomic DNA fragmentation using Covaris M220 Focused Ultrasonicator (Covaris, MA, United States, M220), and an Illumina Hiseq library was constructed using the TruSeq™ DNA Sample Prep Kit (Illumina, San Diego, CA, United States, catalog No. FC-121-2003). Then, bridge PCR was performed using TruSeq PE Cluster Kit v3-cBot-HS (Illumina, San Diego, CA, United States, catalog No. PE-401-3001). Finally, viral metagenomic sequencing was performed using the Truseq SBS Kit v3-HS (Illumina, San Diego, CA, United States, catalog No. FC-401-3001) on the Illumina Novaseq 6000 platform (San Diego, CA, United States).

Sequencing was completed in collaboration with the Origin-gene biology Co., Ltd. (Shanghai, China).

### Data analysis

The Illumina PE250 sequencing first obtained valid sequences from all samples according to the barcodes before paired-end reads from the original DNA fragments were merged using FLASH (version 1.2.11). UPARSE software package (Edgar, 2013) (version 7.0.1090)^1^ was employed for sequencing analysis. Sequences with ≥97% similarity were assigned to the same operational taxonomic units (OTUs), and RDP classifier (Wang et al., 2007) (version 2.2)^2^ Bayesian algorithm was used for the taxonomic analysis of the representatives OTU sequences from the phylum to species. For the diversity analysis of a single sample (alpha diversity), we calculated the number of unique OTUs, community richness marker Chao-the Chao1 estimator,^3^ community diversity marker Shannon-the Shannon index,^4^ Simpson-the Simpson index,^5^ and sequencing depth index coverage-the Good’s coverage^6^ for every sample. Inter-sample diversity analysis (beta analysis) was conducted on the Qiime platform (Caporaso et al., 2010) (version 1.9.0)^7^ and weighted and unweighted unifrac distances were calculated. Bray-Curtis and complete methods in the vegan package of R were applied for distance calculation and clustering analysis, respectively. R was used to calculate the number of common and unique OTUs in multiple samples, as depicted with Venn diagrams (Fouts et al., 2012).

Illumina Hiseq sequencing chromatogram data was converted to sequencing data using the Base Calling software. The Cutadapt^8^ software was used for quality cleavage and the quality control of source data. BWA^9^ was used for the alignment of high-quality data and host genome to eliminate host DNA contamination. The megahit (version 1.1.3)^10^ software was used for *de novo* assembly of the sequencing data. Contigs in the assembled results were applied for the ORF prediction to construct non-redundant gene sets. Next, SOAPaligner (version 2.21)^11^ was used to align high-quality reads of every sample and non-redundant gene sets (95% identity), and the abundant information of genes in corresponding samples was calculated. Then, the blast was used for blastp alignment of protein sequences of non-redundant genes and virus sequences. Finally, the optimal alignment results of every sequence were employed for species taxonomic annotation. BLASTP (BLAST Version 2.2.28 +)^12^ and MEGAN (Huson et al., 2011)^13^ were used to return the species abundance information from sequencing in the NCBI database.

## Results

### Illumina PE250

#### General statistics

##### Sequencing data statistics

After calibration and chimera removal of the sequencing results from 3 samples (SK1, SK2, and SK3), a total of 182,116 (54691, 53493, and 73932, respectively) optimized data sequences were obtained, where the length of this optimized data was 74,265,823 bp (22,156,008, 21,666,667, and 30,443,148 bp, respectively). The mean length of these optimized sequences was 407.79 bp (405.11, 405.04, and 411.77 bp, respectively), and 99.99% of the sequences were mapped to a 401–440-bp region. OTU selection and taxonomic assignments were employed to generate 174,533 (51,972, 51,609, and 70,952, respectively) high-quality, effective clean reads for analysis.

##### Alpha-diversity analysis

A similarity level of 97% was applied to calculate the alpha-diversity indices (Shannon, Simpson, Chao, ACE, and Good’s coverage). The results demonstrated that the SK3 sample had the highest Shannon value and the lowest Simpson value and that the SK1 sample had the highest Chao and Ace indices (Table 1). The Good’s coverage of the three samples exceeded 99.99%.

**TABLE 1:** Microbial richness and alpha-diversity indices of the samples.

Sample name	0.97				
	
	Shannon index	Simpson index	Chao1 estimator	ACE index	Good’s coverage (%)
SK1	0.16	0.94	20	21	99.99
SK2	0.19	0.91	15	16	99.99
SK3	0.85	0.54	16	16	99.99

##### Operational taxonomic unit cluster analysis

**FIGURE 2:**
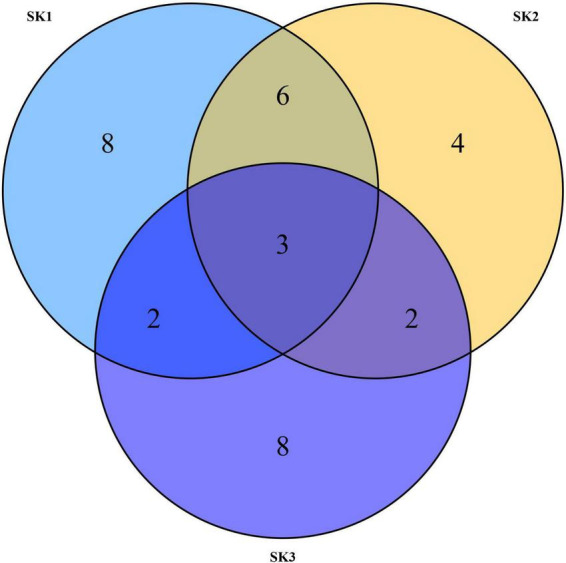
Venn diagrams of three *Melophagus ovinus* samples based on OTUs.

As shown in the Venn diagram in Figure 2, 19, 15, and 15 OTUs were obtained from SK1, SK2, and SK3 samples, respectively. Among these, nine OTUs showed high similarity between SK1 and SK2 samples, five OTUs showed high similarity between SK1 and SK3 samples, five OTUs showed high similarity between SK2 and SK3 samples, and three OTUs showed high similarity in all three samples. In all samples, most tags were classified, of which 97% of the tags were assigned to the genus level. Only a minority of tags were classified at the species level.

#### Microbial population

##### Microbial characteristics at the phyla level

Three phyla were annotated to SK1 and SK2 (Proteobacteria, Firmicutes, and Actinobacteria), and three phyla (Chloroflexi, Cyanobacteria, and Bacteria unclassified) were annotated to SK3. The abundance of Proteobacteria in the three samples was 98.96, 99.91, and 99.84%, respectively, showing a marked predominance. In addition, the relative abundances of Actinobacteria (0.42%), Firmicutes (0.61%) in SK1, and Actinobacteria (0.14%) in SK3 were also high.

##### Microbial characteristics at the genus level

**FIGURE 3:**
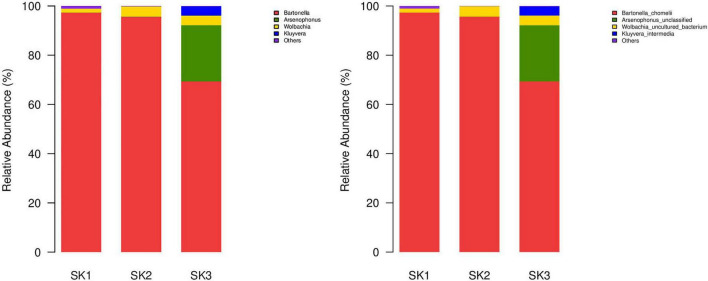
Microbial community bar plot of bacteria from three *Melophagus ovinus* samples (Left: Bacterial genus; Right: Bacterial species).

A total of 29 bacterial genera were annotated in 3 samples, of which 16, 14, and 14 genera were present in SK1, SK2, and SK3 samples, and only 4 genera (*Bartonella*, *Wolbachia*, *Arsenophonus*, and *Corynebacterium* 1) were common among the 3 samples. Eighteen genera (*Acinetobacter*, *Brachybacterium*, Candidatus *Pelagibacter*, *Cellulomonas*, *Glutamicibacter*, JG30-KF-CM45 norank, *Kluyvera*, *Micrococcaceae* unclassified, *Microcoleus*, *Micromonosporaceae* unclassified, *Nocardiopsis*, *Ornithinimicrobium*, *Propioniciclava*, SAR116 clade norank, SAR86 clade norank, *Salinicoccus*, *Staphylococcus*, and ZD0405 norank) were present in only one of the three samples. *Bartonella* and *Wolbachia* were common dominant bacterial genera in three samples, and the relative abundance of *Arsenophonus* was relatively high in SK3 but low in SK1 and SK2. In SK1, the relative abundances of *Corynebacterium* 1, *Jeotgalicoccus*, *Nocardiopsis*, and *Staphylococcus* were high. In SK3, the relative abundances of *Kluyvera* and *Rhodococcus* were high, but the relative abundances of other genera were low (Table 2 and Figure 3 left).

**TABLE 2:** The relative abundance of 29 bacterial genera in the 3 samples.

Bacterial genus	Relative abundance of bacterial genus in each sample (%)		
	
	SK1	SK2	SK3
*Acinetobacter*	0	0	0.0058
*Amylibacter*	0.0097	0.0039	0
*Arsenophonus*	0.0368	0.0581	22.8371
*Bartonella*	97.3086	95.6442	69.3348
*Brachybacterium*	0.0039	0	0
Candidatus *Pelagibacter*	0	0	0.0058
*Cellulomonas*	0	0.0039	0
*Corynebacterium* 1	0.1085	0.0097	0.0078
*Glutamicibacter*	0.0116	0	0
JG30-KF-CM45 norank	0	0	0.0058
*Jeotgalicoccus*	0.1395	0.0019	0
*Kluyvera*	0	0	3.7242
*Micrococcaceae* unclassified	0.0271	0	0
*Microcoleus*	0	0	0.0019
*Micromonosporaceae* unclassified	0	0.0058	0
*Nocardiopsis*	0.2732	0	0
*Ornithinimicrobium*	0	0	0.0058
*Planktomarina*	0.0019	0.0136	0
*Planomicrobium*	0.0213	0	0.0058
*Propioniciclava*	0	0	0.0058
*Rhodobacteraceae* unclassified	0.0019	0.0039	0
*Rhodococcus*	0	0.0601	0.1240
*Romboutsia*	0	0.0039	0.0019
SAR116 clade norank	0	0.0058	0
SAR86 clade norank	0	0.0039	0
*Salinicoccus*	0.0039	0	0
*Staphylococcus*	0.4495	0	0
*Wolbachia*	1.5986	4.1814	3.9295
ZD0405 norank	0.0039	0	0

*Norank (* unclassified) does not have species information at the current classification level and is represented by the known nearest ancestor classification name + norank (unclassified).

##### Microbial characteristics at the species level

At least seven bacterial species were identified in the three samples, of which the predominant bacteria *Ba. chomelii* and *Wolbachia* spp. were common to the three samples, and the relative abundances of *Ba. chomelii* and *Wolbachia* spp. in SK1, SK2, and SK3 were 97.31 and 1.60%, 95.64, and 4.18%, and 69.33 and 3.93%, respectively. *Arsenophonus* spp. was present in three samples, but only SK3 had a high relative abundance (SK1—0.04%, SK2—0.06%, SK3—22.84%) (Figure 3 Right). *Kluyvera intermedia* was only identified in SK3, and its relative abundance (3.72%) was also high. *Corynebacterium maris* DSM 45190 (0.09, 0.002%) were also identified in the SK1 and SK2 samples. *Planomicrobium okeanokoites* (0.02, 0.01%) were also identified in the SK2 and SK3 samples. *Rhodococcus erythropolis* (0.06, 0.12%) were also identified in the SK2 and SK3 samples, but their relative abundances were low.

### Illumina hiseq sequencing results

A total of 162,989,660 reads were obtained from the three samples (SK1: 62474042, SK2: 42703260, and SK3: 57812358) on the Illumina Novaseq 6000 platform. After quality control, the raw data was 104,774,210 reads (37,614,416, 29,461,180, and 37,698,614, respectively) and GC% was 35.21, 33.45, and 33.17, respectively. After *de novo* assembly of the quality control data, the assembled genome sequences were obtained. After ORF prediction, 2,156,816 reads (5.7340%, 2,156,816/37,614,416), 1,377,322 reads (4.67504%, 1,377,322/29,461,180), and 2,156,990 reads (5.7217%, 2,156,990/37,698,614) were annotated as viruses, respectively.

Four identical virus orders were annotated in the three samples (except for Caudovirales, the virus gene prediction reads were mainly annotated as o__norank_d__Viruses, and a few reads were annotated as Herpesvirales and Ortervirales, Table 3). The 3 samples were, respectively, annotated into 29 families (corresponding to 4 aforementioned orders: 5 families, 17 families, 4 families, and 3 families), 30 families (corresponding to 4 aforementioned orders: 5 families, 18 families, 4 families, and 3 families), and 28 families (corresponding to 4 aforementioned orders: 5 families, 17 families, 3 families, and 3 families). In o__norank_d__Viruses, 3 samples were annotated into 16 common families (f__norank_d__Viruses, Mimiviridae, Phycodnaviridae, Poxviridae, Pithoviridae, Inoviridae, Baculoviridae, Polydnaviridae, Marseilleviridae, Iridoviridae, Nudiviridae, Adomaviridae, Geminiviridae, Fuselloviridae, Hytrosaviridae, Asfarviridae), Hepeviridae was found in SK1 and SK2, Ascoviridae was found in SK3, and Microviridae was found in SK2. Among o__norank_d__Viruses, the number of annotated reads of certain families in one of the three samples that accounted for >1% of annotated reads were in four identical families, namely f__norank_d__Viruses (22.10, 23.57, and 23.89%, respectively), Mimiviridae (13.47, 13.78, and 12.51%, respectively), Phycodnaviridae (9.17, 9.79, and 8.27%, respectively), and Poxviridae (1.55, 1.51, and 1.44%, respectively). Herpesviridae had the highest number of annotated reads in Herpesvirales in all three samples (1492, 860, and 414, respectively), and Retroviridae had the highest number of annotated reads in Ortervirales (5856, 6538, and 4462, respectively).

**TABLE 3:** Statistical table for the number of reads of three samples at the order level.

Order	Caudovirales			o__norank_d__Viruses			Herpesvirales			Ortervirales		
				
Sample name	SK1	SK2	SK3	SK1	SK2	SK3	SK1	SK2	SK3	SK1	SK2	SK3
Precent (Reads_number)	51.49% (1110588/ 2156816)	48.11% (662646/ 1377322)	52.11% (1124024/ 2156990)	47.96% (1034390/ 2156816)	51.01% (702600/ 1377322)	47.62% (1027184/ 2156990)	0.09% (1994/ 2156816)	0.08% (1168/ 1377322)	0.02% (440/ 2156990)	0.28% (6048/ 2156816)	0.49% (6744/ 1377322)	0.28% (4678/ 2156990)

There is no scientific name for this level in the taxonomic pedigree, and it is marked with norank.

**FIGURE 4:**
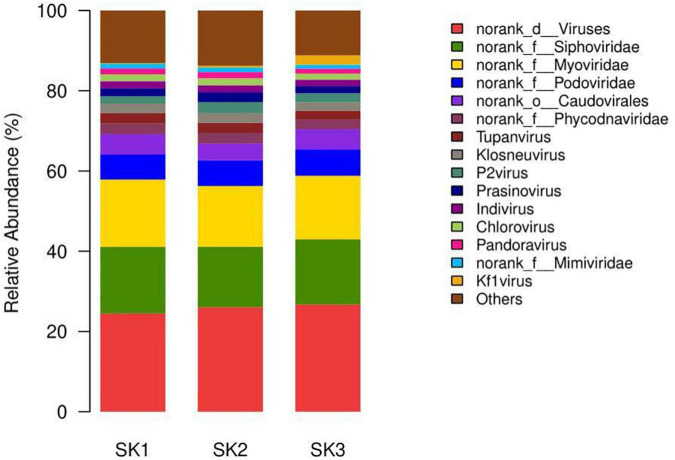
The microbial community bar plot of virus genus of the three *Melophagus ovinus* samples.

At the genus level, genera with species transcripts per million (TPM) abundance to the sample’s total abundance ≥1% (Table 4) include six genera in four families in Caudovirales and also included two genera from the f__norank_d__Viruses family (g__norank_d__Viruses and Pandoravirus), three genera from the Phycodnaviridae family (norank_f__Phycodnaviridae, Prasinovirus, Chlorovirus), and four genera from the Mimiviridae family (Tupanvirus, Klosneuvirus, Indivirus, norank_f__Mimiviridae) (Figure 4).

**TABLE 4:** Transcripts per million (TPM) abundance percentage of samples at the genus level.

Taxon			Abundance percentage of genus level (%)		
	
Order	Family	Genus	SK1	SK2	SK3
Caudovirales	Siphoviridae	norank_f__Siphoviridae	16.60	15.06	16.22
	norank_Caudovirales	norank_o__Caudovirales	5.07	4.20	5.06
	Myoviridae	norank_f__Myoviridae	16.80	15.13	15.86
		P2virus	1.81	2.77	2.24
	Podoviridae	norank_f__Podoviridae	6.21	6.38	6.56
		Kf1virus	0.17	0.46	2.37
o__norank_d__Viruses	f__norank_d__Viruses	g__norank_d__Viruses	24.48	26.07	26.73
		Pandoravirus	1.45	1.54	1.23
	Phycodnaviridae	norank_f__Phycodnaviridae	2.67	2.67	2.41
		Prasinovirus	1.97	2.42	1.75
		Chlorovirus	1.74	1.72	1.52
	Mimiviridae	Tupanvirus	2.55	2.48	2.16
		Klosneuvirus	2.42	2.41	2.13
		Indivirus	1.78	1.77	1.63
		norank_f__Mimiviridae	1.15	1.14	0.96

There is no scientific name for this level in the taxonomic pedigree, and it is marked with norank.

At the species level, those with TPM abundance ≥1% of the sample’s total abundance (Table 5) included 12 species, 5 genera, and 4 families in Caudovirales and 7 species in the g__norank_d__Viruses genus in the f__norank_d__Viruses family. In addition, it included three species and three genera from the Mimiviridae family: Tupanvirus (Tupanvirus_soda_lake), Klosneuvirus (Klosneuvirus_KNV1), and Indivirus (Indivirus_ILV1). Furthermore, more viruses were simultaneously detected in the three samples. Although the relative abundances were low, these pathogens could cause common and serious diseases to some livestock, such as the African swine fever virus and different Poxviruses in Poxviridae.

**TABLE 5:** Transcripts per million (TPM) abundance percentage of samples at the species level.

Taxon				Abundance percentage of Species level		
	
Order	Family	Genus	Species	SK1	SK2	SK3
Caudovirales	Siphoviridae	norank_f__Siphoviridae	Bacteroides_phage_B124-14	3.00	2.30	3.32
			Pseudomonas_phage_PS-1	1.20	1.34	0.91
			Paracoccus_phage_Shpa	1.10	1.38	1.35
	norank_Caudovirales	norank_o__Caudovirales	Aurantimonas_phage_AmM-1	2.59	1.98	2.76
	Myoviridae	norank_f__Myoviridae	Bacillus_virus_G	3.80	3.42	3.54
			Myoviridae_sp.	2.51	2.30	2.41
			Agrobacterium_phage_Atu_ph07	1.60	1.50	1.57
			Bacillus_phage_SP-15	1.00	0.88	0.91
		P2virus	Salmonella_phage_SEN5	0.92	1.22	1.06
			Burkholderia_virus_phiE122	0.87	1.28	1.17
	Podoviridae	norank_f__Podoviridae	Sinorhizobium_phage_PBC5	1.66	1.34	1.71
			Klebsiella_phage_SopranoGao	0.77	1.03	0.94
o__norank_d__Viruses	f__norank_d__Viruses	g__norank_d__Viruses	uncultured_Mediterranean_phage	4.06	3.76	3.91
			uncultured_Mediterranean_phage_uvMED	3.62	3.88	3.76
			Wolbachia_phage_WO	3.05	4.44	4.84
			Bartonella_henselae_phage_60457	2.76	2.43	2.65
			Sulfitobacter_phage_pCB2047-C	1.11	1.29	1.20
			Wolbachia_phage_WOcauB1	0.82	1.11	1.35
			Sinorhizobium_phage_phiM6	0.77	1.17	1.05
	Mimiviridae	Tupanvirus	Tupanvirus_soda_lake	1.57	1.57	1.35
		Klosneuvirus	Klosneuvirus_KNV1	1.95	1.89	1.75
		Indivirus	Indivirus_ILV1	1.78	1.77	1.63

There is no scientific name for this level in the taxonomic pedigree, and it is marked with norank. Environmental sequences that were not isolated and cultured were labeled as uncultured.

## Discussion

From 1950 to 2018, 124 tick species, 103 tick-borne agents, and 29 tick-borne pathogens (Zhao et al., 2021) infecting humans have been reported in China. In the 33 years since 1982, 33 emerging tick-associated agents have been identified in mainland China, of which 20 have been confirmed to cause human disease (Fang et al., 2015). With regards to pathogen carriage and transmission, various species of mosquitoes and ixodid ticks have been actively studied (Litov et al., 2021). More than 213 species belonging to 21 genera (Liu et al., 2017; Werszko et al., 2021) in the family Hippoboscidae (Diptera) were reported globally, but less attention has been paid to these insects. Among them, *M. ovinus* has been the most studied species relatively. Undoubtedly, advances in molecular technologies and their applications have led to the discovery of novel agents. However, most studies on *M. ovinus* are limited to the detection of specific pathogens. High-throughput sequencing is expedient and efficient for the analysis of the microbial community structure of *M. ovinus*. However, high-throughput sequencing has only been conducted on microbial population and RNA virome in *M. ovinus* from the Gansu province in China (Duan et al., 2017, 2020) and the Republic of Tuva in Russia (Litov et al., 2021) so far. Several factors such as biogeography, the season of sample collection, and the number of samples may induce differences in pathogenic microorganisms carried by *M. ovinus* in different regions (Zhang et al., 2021). In this study, we employed high-throughput sequencing to analyze the microbial community structure and DNA viruses in *M. ovinus* from three regions in Tibet, China.

In 16S rRNA V3-V4 high-throughput sequencing, a greater Shannon index and a lower Simpson index indicate higher microbial community diversity of the sample. A higher Ace value indicates that the total number of species is higher (Duan et al., 2017, 2020). The sequencing results showed that the SK3 sample had the highest microflora diversity and the SK1 sample had the highest total number of species. The sequencing depth Good’s coverage of the three samples all exceeded 99.9%, indicating that the sequencing depth was sufficient to demonstrate microbial diversity of the samples and reflected the actual status of microorganism carriage in the samples. OTU cluster analysis revealed that some microbial populations were identical between the samples. On the basis of the aforementioned estimators, an analysis of the sequencing data revealed that Proteobacteria is a dominant bacterial phyla common in the three *M. ovinus* samples from Tibet, China, which is consistent with the *M. ovinus* results reported from Gansu province, China (Duan et al., 2017, 2020). Twenty-nine bacteria genera were annotated in *M. ovinus* from Tibet, China, which included six bacteria genera reported in the past *M. ovinus* studies (*Bartonella*, *Wolbachia*, *Arsenophonus*, *Staphylococcus*, *Acinetobacter*, and *Salinicoccus*). Among them, 23 bacteria genera (*Amylobacter*, *Brachybacterium*, Candidatus *Pelagibacter*, *Cellulomonas*, *Corynebacterium* 1, *Glutamicibacter*, JG30-KF-CM45 norank, *Jeotgalicoccus*, *Kluyvera*, *Micrococcaceae* unclassified, *Microcoleus*, *Micromonosporaceae* unclassified, *Nocardiopsis*, *Ornithinimicrobium*, *Planktomarina*, *Planomicrobium*, *Propioniciclava*, *Rhodobacteraceae* unclassified, *Rhodococcus*, *Romboutsia*, SAR116 clade norank, SAR86 clade norank, ZD0405 norank) were not previously reported in *M. ovinus*. *Bartonella* and *Wolbachia* were dominant bacterial genera common to the three samples. *Arsenophonus* was the dominant bacterial genus of SK3, where the dominant bacterial genera were also consistent with the results of *M. ovinus* reported in the Gansu province, China (Duan et al., 2017, 2020). The relative abundances of bacterial genera *Corynebacterium* 1, *Jeotgalicoccus*, *Kluyvera*, *Nocardiopsis*, and *Staphylococcus* in *M. ovinus* from Tibet, China were also high, particularly that of *Kluyvera*. At the species level, the reported *Ba. chomelii*, *Wolbachia* spp., and *Arsenophonus* spp. were identified. In addition, *K. intermedia*, *Corynebacterium maris* DSM 45190, *Planomicrobium okeanokoites*, and *Rhodococcus erythropolis* were discovered for the first time. As per the literature (Liu et al., 2016) and our previous study (unpublished), the positivity rate of *Rickettsia* in *M. ovinus* in Xinjiang province, China is also high. Strangely, *Rickettsia* was not detected in *M. ovinus* from Tibet in this study, implying that the Rickettsia carriage in *M. ovinus* could be based on the geographical region.

*Bartonella* mainly infects mammals and causes intraerythrocytic bacteremia. At least 30 *Bartonella* species have been reported to cause salonica fever, cat-scratch fever, and Carrion’s disease in humans (Duan et al., 2017, 2020). The main vectors are blood-sucking parasitic arthropods, such as ticks, chiggers and lice (Duan et al., 2017). In 2004, the presence of *Bartonella* in ruminant blood-sucking flies (*Lipoptena cervi*, *Hippobosca equina*, *M. ovinus*) was first reported. *Bartonella* can spread through hematophagous arthropods and is considered a symbiont in some insects, of which *M. ovinus* is a potential vector (Halos et al., 2004; Duan et al., 2020). *Bartonella* is common in *M. ovinus*, and several *Bartonella* species were previously identified in *M. ovinus*. However, *Ba. melophagi* is the most common (Halos et al., 2004; Kosoy et al., 2004, 2016; Reeves et al., 2006; Bemis and Kania, 2007; Kumsa et al., 2014; Rudolf et al., 2016; Liu et al., 2018; Boucheikhchoukh et al., 2019; Duan et al., 2020; Flores-Mendoza et al., 2021; Werszko et al., 2021). *Ba. chomelii* was first discovered in French domestic cows and is the 4th *Bartonella* species to be isolated from the ruminants. The species was named in honor of Bruno B. Chomel (Maillard et al., 2004). *Ba. chomelii* is the most common species of beef cattle infection in the Basque Country in Spain (Antequera-Gómez et al., 2015). *Ba. chomelii* was first identified in *M. ovinus* from the Gansu province, China, and is a dominant bacterial species. It has also been reported that the abundance of *Ba. chomelii* in fully engorged adult female samples (46.5 and 16.4%) was significantly higher than that in newly hatched and unfed adult female samples (1.19 and 1.17%, respectively), where the proliferation of *Ba. chomelii* was enhanced eventually with blood-feeding (Duan et al., 2020). This was consistent with the test results of engorged *M. ovinus* in Tibet, China, in this study, and the relative abundances of the three samples were 97.31, 95.64, and 69.33%, respectively. Considering the aforementioned reasons, it is necessary to further examine the specific role of *Ba. chomelii*, with high abundance, in *M. ovinus*, and the risk of transmission of *M. ovinus*-derived and ruminant-derived *Bartonella* to humans should not be underestimated.

Wolbachia is an obligate intracellular bacterium that can infect many hosts, such as ticks, sand flies, tsetse flies, mosquitos, fleas and mites, and cause reproductive manipulation and parthenogenesis through cytoplasmic incompatibility, thereby regulating reproductive behavior (Duan et al., 2017, 2020). Previously, it was reported that *M. ovinus* carries Wolbachia (Duan et al., 2017, 2020; Liu et al., 2018; Boucheikhchoukh et al., 2019), and Wolbachia is also a common dominant bacterial genus among the three samples. However, the role of Wolbachia remains to be determined. Arsenophonus is an intracellular symbiotic bacterium with a broad host range and infects several arthropods (Duan et al., 2017). Arsenophonus is also present in *M. ovinus* (Nováková et al., 2015; Duan et al., 2017, 2020; Husnik et al., 2020), which plays an important role in killing male hosts and provides vitamins and other nutrients to host insects (Duan et al., 2017). Further studies are required to determine the role of Arsenophonus in *M. ovinus* and to understand whether it is related to nutrient supply. A more challenging question is whether Wolbachia and Arsenophonus should be considered endosymbionts or sheep parasites of *M. ovinus*. The answer to this question is not straightforward and requires confirmation through a large number of experiments by researchers.

*Kluyvera intermedia* was previously reported to be isolated from patients (Thele et al., 2017; Paskova et al., 2018), and *K. intermedia* originating from spiders has been reported to be pathogenic to humans (Dunbar et al., 2020). The genus Kluyvera has the potential to act as a rare infectious agent and causes soft tissue infections, urinary tract infections, intra-abdominal abscesses, catheter-associated bloodstream infections and septic shock (Thele et al., 2017). This study was the first to identify *K. intermedia* in *M. ovinus*, with a relatively high abundance. The risk of *K. intermedia* transmission by *M. ovinus* to sheep or humans is worthy of close attention by researchers.

The results of viral metagenomic sequencing revealed that all three samples were annotated to four identical virus orders (Caudovirales, o__norank_d__Viruses, Herpesvirales, and Ortervirales). At the family level, after excluding phage-related Caudovirales, which was not the focus of this study, viruses associated with vertebrates and insects that were annotated in o__norank_d__Viruses included Mimiviridae, Marseilleviridae, Poxviridae, Ascoviridae, Iridoviridae, Baculoviridae, Hytrosaviridae, Nudiviridae, Polydnaviridae, Adomaviridae, Asfarviridae, Hepeviridae, and some plant viruses and algal viruses were annotated. The three samples were mainly annotated to Herpesviridae and Retroviridae in Herpesvirales and Ortervirales, respectively. The abovementioned viruses were not reported in *M. ovinus* in Diptera. Mimiviridae is one of the largest and most diverse families of eukaryotic viruses isolated from aquatic environments. This family of viruses can infect a broad range of eukaryotic unicellular organisms, ranging across six major phyla: Amoebozoa, Chlorophyta, Haptophyta, Heterokonta, Excavata, and perhaps Opisthokonta (Claverie and Abergel, 2018). In this family, we identified three genera with high abundance, namely Tupanvirus, Klosneuvirus, and Indivirus. The family Marseilleviridae is a taxon for giant viruses that infect amoeba, where this family along with the family Mimiviridae, as well as the families Poxviridae, Iridoviridae, Ascoviridae, Asfarviridae all belong to a viral family among nucleo-cytoplasmic large DNA viruses (Colson et al., 2013). This list includes the genus *Pandoraviruses* with a high abundance, which was annotated to the f__norank_d__Viruses family—giant viruses that infect amoeba (Takemura, 2020). It is still unknown whether these annotated giant viruses are related to protozoal infection in *M. ovinus*. Currently, large dsDNA viruses that are known to circulate in insects include Poxviridae, Ascoviridae, Iridoviridae, Baculoviridae, Hytrosaviridae, Nudiviridae, where Iridoviruses, Poxviruses, and Nudiviruses are ubiquitous in several insect orders; Baculoviruses is reported to be present in Lepidoptera, Hemiptera, and Diptera; and Ascoviruses infects lepidopteran larvae (Gilbert and Belliardo, 2022). The Hytrosaviruses (family: Hytrosaviridae) are viruses identified in different dipteran species. The family includes two genera (Muscavirus and Glossinavirus). Hytrosaviruses can cause salivary gland hypertrophy and testicular and ovarian malformation leading to sterility in adult dipterans. Currently identified Hytrosaviruses only include three virus species from the three genera of Diptera: the tsetse fly *Glossina pallidipes*, the housefly *Musca domestica*, and the narcissus bulb fly *Merodon equestris* (Abd-Alla et al., 2009; Lacey, 2012; Omkar, 2016; Kariithi et al., 2017). Two Hytrosaviridae genera, Muscavirus and Glossinavirus were detected in our three samples as well as other insect-related virus families Poxviridae, Iridoviridae, Baculoviridae, and Nudiviridae. Insects-associated viruses Ascoviridae were also detected in the SK3 samples. The virus families Polydnaviridae, Asfarviridae, Herpesviridae, and Retroviridae were annotated in three samples, Hepeviridae was annotated in two samples, and several viruses were annotated in Poxviridae. Although the abundances of some annotated viruses are low, their presence is not a complete coincidence. This is the first discovery of these viruses in *M. ovinus* from the order Diptera. Particularly, Hytrosaviridae could be a potential biocontrol agent, warranting further in-depth research. In addition, adomaviruses, adenoviruses, papillomaviruses, parvoviruses, and polyomaviruses are collectively known as small DNA tumor viruses. Small DNA tumor virus oncoproteins and capsid proteins demonstrated structural and functional similarity (Welch et al., 2019). Further evaluation is warranted to examine whether these viruses can proliferate in *M. ovinus*, play a role in pathogen dissemination in *M. ovinus* and are involved in the risk of causing human diseases. Finally, we would like to mention one of the many viruses that were annotated in three samples—the African swine fever virus (ASFV). In 2018, an outbreak of African swine fever (ASF) occurred in China, which was the site of the first outbreak of Asian ASF (Abd-Alla et al., 2009). Countries that experienced ASF outbreaks had to undergo catastrophic pig culling. More importantly, soft ticks are biological transmission vectors of ASFV (Cheng and Ward, 2022). It has been previously reported that *M. ovinus* is a mechanical vector for the blue-tongue virus (Luedke et al., 1965) and that *M. ovinus* may or may not be a biological or mechanical vector for ASFV.

Several microorganisms colonize in insect vectors, including endosymbionts, potentially pathogenic pathogens, and non-pathogenic pathogens. Microorganisms in insect vectors play important roles in pathogen survival, transmission, modulating vector competence, reproductive fitness, and nutrient supply (Duan et al., 2020). Evaluating microorganisms in insect vectors could help in the further study on how microorganisms interact with vectors of animal parasite and on ways to develop biological control tools. *M. ovinus* is a blood-sucking insect that was directly collected from sheep in our study. This finding implies that microorganisms found in this study may infect *M. ovinus* or sheep or circulate between *M. ovinus* and sheep (as an insect-borne pathogen). This study found that several pathogens are present in other insects, other species in Diptera, or *M. ovinus*, implying that some of the pathogens found in this study may be specific to arthropods. However, the possibility of these pathogens being sheep pathogens or insect-borne pathogens cannot be ignored. Although we noted the diversity of microorganisms carried by *M. ovinus*, the report may be the tip of the iceberg with regards to the actual number of pathogens carried and transmitted by *M. ovinus*. In addition, similar to the long period needed to confirm human infection after discovering potential pathogenic factors in ticks, tick-borne pathogens that infect livestock may ultimately cause human disease (Fang et al., 2015). Therefore, we require a large volume of studies to demonstrate the pathogens carried by *M. ovinus* and the pathogenicity of these pathogens. In summary, we mined microorganisms in *M. ovinus* and expanded the spectrum of pathogens that could be present in *M. ovinus*. Our study has veterinary and medical significance and can facilitate prediction of the emerging pathogens, evaluation of potential risks to animal and public health, development of biological control tools, and control management of insect-borne diseases.

## Conclusion

Proteobacteria is the dominant bacterial phylum in *M. ovinus* from Tibet, China, and 29 bacteria genera were annotated. The dominant bacterial genera included *Bartonella*, *Wolbachia*, and *Arsenophonus* and this is the first report of 23 bacteria genera in *M. ovinus*. In addition, this is also the first report of *Kluyvera intermedia*, *Corynebacterium maris* DSM 45190, *Planomicrobium okeanokoites*, and *Rhodococcus erythropolis*. All DNA viruses reported in this study are the first to be reported in *M. ovinus*.

## Data availability statement

The datasets presented in this study can be found in online repositories. The names of the repository/repositories and accession number(s) can be found below: The raw tags have been deposited in Sequence Read Archive (SRA) from the NCBI under BioProject accession numbers: PRJNA849748 and PRJNA852367. The individual run files received the accession numbers SRR19668428, SRR19668429, SRR19668430, SRR19834166, SRR19834167, and SRR19834168.

## Ethics statement

The animal study was reviewed and approved by the biomedical research ethics committee of Inner Mongolia Agricultural University specifically approved this study [No. 2020 (080)]. Written informed consent was obtained from the owners for the participation of their animals in this study.

## Author contributions

Y-HL and LZ conceived and designed the study and critically revised the manuscript. Y-LD, LZ, BY, and W-XH performed the sheep ked collection. Y-HL, Y-MM, H-OT, W-HZ, H-LC, Z-SZ, L-FW, and Y-LD conducted the laboratory experiments. Y-HL, LZ, Y-MM, YX, and LC conducted sequencing and participated in sequence analysis. All authors read and approved the final manuscript.

## Footnotes

1
http://drive5.com/uparse/

2
http://sourceforge.net/projects/rdp-classifier/

3
http://www.mothur.org/wiki/Chao

4
http://www.mothur.org/wiki/Shannon

5
http://www.mothur.org/wiki/Simpson

6
http://www.mothur.org/wiki/Coverage

7
http://qiime.org/scripts/assign_taxonomy.html

8
https://cutadapt.readthedocs.io/en/stable/

9
http://bio-bwa.sourceforge.net/

10
http://i.cs.hku.hk/~alse/hkubrg/projects/idba_ud/

11
https://github.com/gigascience/bgi-soap1

12
http://blast.ncbi.nlm.nih.gov/Blast.cgi

13
http://ab.inf.uni-tuebingen.de/software/megan/
